# Effects of low‐dose *Aloe* sterol supplementation on skin moisture, collagen score and objective or subjective symptoms: 12‐week, double‐blind, randomized controlled trial

**DOI:** 10.1111/1346-8138.15428

**Published:** 2020-06-08

**Authors:** Chikako Kaminaka, Yuki Yamamoto, Mariko Sakata, Chiaki Hamamoto, Eriko Misawa, Kazumi Nabeshima, Marie Saito, Miyuki Tanaka, Fumiaki Abe, Masatoshi Jinnin

**Affiliations:** ^1^ Department of Dermatology Wakayama Medical University Graduate School of Medicine Wakayama Japan; ^2^ Functional Food Ingredients Department Food Ingredients and Technology Institute Morinaga Milk Industry Co., Ltd Zama Japan

**Keywords:** adverse events, collagen, hydration, moisture, ultrasound

## Abstract

Daily oral intake of 40 μg *Aloe* sterol was shown in a double‐blind clinical trial to significantly increase skin barrier function, moisture and elasticity. Ultrasonographic results also suggested that the intake of *Aloe* sterol increases collagen content in the dermis. Here, we evaluate the effects of a much smaller dose of *Aloe* sterol, approximately half that used previously, on skin functions in more detail. This is a monocentric, double‐blind, randomized, placebo‐controlled, supplementation study of the effects of low‐dose *Aloe* sterol on skin transepidermal water loss, hydration, collagen score, evaluation of objective or subjective symptoms, and safety after 12 weeks of daily intake. We randomly administrated either *Aloe* sterol or placebo to 122 healthy volunteers. Transepidermal water loss was significantly reduced and collagen score was increased in the *Aloe* sterol group compared with the placebo group at week 12. In the *Aloe* sterol group, there was significant improvement of objective skin condition (face erythema and pruritus of inner and outer arms) at week 12 compared with week 0, but not in the placebo group. Subjectively, there was significant improvement of visual analog scale of skin acne, fingernail brittleness and constipation in the *Aloe* sterol group. According to subgroup analysis, although not planned before the study initiation, subjects with dry skin in the *Aloe* sterol group had significantly increased skin hydration values at week 12 compared with the placebo group. Our results confirmed that even low‐dose *Aloe* sterol ingestion improves skin moisture by promoting skin barrier function and dermal collagen production, which contributes to maintenance of healthy skin.

## INTRODUCTION

Skin dryness is the most common skin problem, and can cause rough skin and increase the risk of developing various skin diseases, such as asteatotic eczema, atopic dermatitis, irritant contact dermatitis and allergic contact dermatitis, which reduce quality of life.^1^, ^2^ Water is essential for the normal functioning of the skin, especially the stratum corneum (SC), its outer layer, and skin moisture is determined mainly by two factors: the water‐holding capability of the SC and the skin barrier function.^3^ Protecting the skin barrier function and retaining skin moisture are therefore important for skin health.^4^, ^5^

To assess the skin moisture condition, several non‐invasive instruments have been developed. For example, the water content at the skin surface can be evaluated by measuring capacitance or impedance.^6^ Skin barrier function can also be determined by electrically measuring the water flux at the surface of the skin, as transepidermal water loss (TEWL). Skin moisture can therefore usually be rated according to parameters of the skin surface water content and TEWL measured with such equipment.

*Aloe barbadensis Miller* (*Aloe vera*) is a tropical plant belonging to the family Liliaceae. The gel of *A. vera*, obtained from inner thin‐walled parenchymal cells, has a long history of being added to health foods, beverages, pharmaceuticals and cosmetics.^7^, ^8^ *A. vera* gel or the mucilaginous portion of *A. vera* contains many kinds of pharmacologically active ingredients.^9^ Acemannan, for example, the major polysaccharide of *A. vera* gel, has immunomodulating function and stimulates proliferation of gingival fibroblasts.^10^, ^11^ Aloesin, a kind of chromone, inhibits melanin synthesis by reducing tyrosinase activity,^12^ and it improves insulin intolerance.^13^ In our previous study, we attempted to isolate hypoglycemic ingredients from *A. vera* gel, and succeeded in the identification of five minor plant sterols (lophenol, 24‐methyl‐lophenol, 24‐ethyl‐lophenol, cycloartanol and 24‐methylene‐cycloartanol) named *Aloe* sterols.^14^ We also demonstrated that *Aloe* sterols work as ligands for peroxisome proliferator‐activated receptors, ligand‐activated transcriptional factors belonging to the nuclear receptor superfamily.^15^

In addition, *A. vera* is popularly utilized for skin care, such as wound healing, sun protection and anti‐aging. Oral supplementation with *A. vera* gel was reported in clinical trials to improve facial wrinkles and elasticity.^16^ We also previously showed that human dermal fibroblasts produced 2‐ and 1.5‐fold levels of collagen and hyaluronic acid after cultivation in the presence of *Aloe* sterol *in vitro*, respectively, and we observed a reduction of the facial wrinkle depth by 8 weeks of oral ingestion of 40 μg *Aloe* sterol in a human study.^17^ Furthermore, we confirmed the presence of statistically significant differences in the levels of skin hydration, TEWL, skin elasticity and the dermal collagen score between 40 μg of the *Aloe* sterol‐treated group and placebo group.^18^ Therefore, the oral ingestion of 40 μg or more of *Aloe* sterol is likely to have beneficial effects on skin functions.

In the current study, we therefore evaluate the effects of a lower dose (approximately half) of *Aloe* sterol (19 μg) on skin functions in healthy female volunteers. Furthermore, we added evaluation of objective or subjective symptoms, and safety monitoring.

## METHODS

### Study design

This is a monocentric, double‐blind, randomized, placebo‐controlled, supplementation study on the effects of *Aloe* sterol on skin function. The primary end‐point of the study is its effect on skin TEWL, hydration and skin elasticity. Secondary end‐points are various skin parameters, subjective symptoms (including profile of mood states [POMS]) and safety monitoring evaluated before and after 12 weeks of daily intake.

The study protocol was conducted according to the guidelines of the Declaration of Helsinki, and approved by the institutional review board of the Japan Conference of Clinical Research (Tokyo, Japan) before initiation of the study. This trial was registered in the UMIN Clinical Trial Registry on 23 November 2018 (UMIN000034963), and performed from December 2018 to March 2019, which corresponds with winter to spring seasons in Japan.

Written informed consent was obtained from all participants before beginning the study, and they were free to withdraw from the study at any time without obligation. The study participants, investigators, staff members and laboratory technicians were all blinded to group assignments.

### Test products

*Aloe vera* gel extract (AVGE) including hydrophobic *Aloe* sterols was obtained by supercritical carbon dioxide extraction from dried mesophyll parts of *A. vera* gel,^19^ and emulsified AVGE powder was prepared by Morinaga Milk Industry (Tokyo, Japan).

*Aloe* sterols comprised two different components with common structures, namely Lop (lophenol, 24‐methyl‐lophenol and 24‐ethyl‐lophenol) and Cyc (cycloartanol and 24‐methylene‐cycloartanol). The test foods contained 8 μg of Lop and 11 μg of Cyc in two capsules.

For this study, two types of capsules were produced under established controlled conditions as test foods, and were packaged in an aluminum bag. The nutrient composition per two capsules was as follows: energy, 2.0 kcal; fat, 0.02 g; protein, 0.00 g; and carbohydrates, 0.56 g. In the *Aloe* sterol capsule, 0.25 g of *Aloe* sterol‐containing AVGE powder was added. In the placebo capsule, the AVGE powder was replaced with starch, and contained no lophenol or cycloartanol.

### Participants

Healthy adult female volunteers ranging in age from 30 to 55 years based on the previous study^18^ were recruited for this study (Table 1). From 177 candidates, individuals who met the exclusion criteria listed in Table 1 and Table S1 were excluded, and 122 participants were enrolled. They might have had minor skin problems including dryness/scales or acne, but did not require medical treatment.

**Table 1:** Criteria for inclusion and exclusion

Inclusion criteria
Japanese adult women aged 30–54 years at the time of enrollment
Exclusion criteria
Individuals who: Regularly use cosmetology or food affecting the skin condition Have skin diseases or diseases affecting skin condition, and/or whose skin has abnormality requiring treatment Need to wear masks or to blow their nose due to allergic rhinitis, and/or may develop the allergy during the study Have chronic diseases Have serious disorders and/or past histories of serious disorders Excessively take alcohol Smoke (>20 cigarettes/day) Have history of serious drug allergy and/or serious food allergy Are pregnant/lactating, and/or desire to/likely become pregnant during the study period Are participating in other clinical trials, and/or participated in other clinical trials within 1 month Were judged to be unsuitable for enrollment based on the opinion of the principal investigator, subject background or physical findings

To note, “Regularly use cosmetology or food affecting the skin condition” assumes aesthetic, laser irradiation and intake of hyaluronic acids or collagen, but did not exclude use of other cosmetics and health foods. To avoid the influence of cosmetics, measurement sites for various evaluation described below included the inner forearm. The subjects were asked to maintain a regular lifestyle and not to use body cosmetics such as lotions during the study period.

### Study schedule and protocol

The study consisted of a 2‐week observation period, followed by a 12‐week intake period. The 122 selected participants were randomly assigned to either the placebo or *Aloe* sterol group. Two participants withdrew before the test food intake period, so 120 subjects (60 individuals/group) participated in this study. Each participant was identified by a code which was randomly selected using a computer‐generated permutation procedure. The code was sequentially allocated to the participants in the order they were enrolled. After all of the measurements were completed, the randomization codes were disclosed to the investigators.

All subjects ingested two capsules per day (*Aloe* sterol or placebo) for 12 weeks. At the baseline (screening examination), every 4 weeks (weeks 4 and 8) and at the end of the intervention (week 12), skin hydration, TEWL and bodyweight were measured. A questionnaire survey about subjective symptoms using a horizontal visual analog scale (VAS) and emotional states using POMS was also performed at each measurement. The dermal collagen score was measured only at the baseline and week 12. At weeks 0 and 12, medical examinations and inquiries were also performed, and a medical doctor assessed objective skin findings. During the study, each subject maintained a daily record of capsule intake, alcohol drinking, any consumption of supplements or forbidden foods, and skin or physical conditions including any subjective symptoms.

### Evaluation of skin parameters

Skin hydration and TEWL were measured on the inner forearm of the non‐dominant hand (test area). On each measurement day, the subjects had to expose their uncovered test area to standard conditions of room temperature (20–22°C) and humidity (45–55%) for at least 20 min before the measurement, to acclimate to the room conditions. Assessment of skin surface hydration by electrical capacitance was carried out using a Corneometer CM825 device (Courage + Khazaka Electronics, Cologne, Germany). The TEWL (g/m^2^ per h) as a marker of the epidermal skin barrier function was detected using a Tewameter TM300 (Courage + Khazaka Electronics) for each site by continuous data logging for a 60‐s period. Analysis of these skin parameters was performed five times at each time‐point, and the mean value was recorded.

Ultrasonography evaluations were performed using the Dermalab skin analysis system (Cortex Technology, Hadsund, Denmark)^18^. The intensity of the reflected echoes was evaluated using a microprocessor and visualized as a colored image. The color scale of echogenicity was as follows: white > yellow > red > green > blue > black. In each image, the collagen score was measured using image analysis software (Cortex Technology).

### Objective clinical assessments by dermatologist

For medical examination and inquiries, a dermatologist assessed skin conditions of participants at 0 and 12 weeks. The severity of wrinkles, dryness/scales, pruritus, erythema and papules were evaluated and scored in the face, inner arms and outer arms. The degree of facial wrinkles was graded as follows: 0, none; 1, slight unclear shallow wrinkles; 2, slight clear shallow wrinkles; 3, clear shallow wrinkles; 4, clear shallow wrinkles and slight deep wrinkles; 5, mild deep wrinkles; 6, moderate deep wrinkles; and 7, remarkable deep wrinkles. The degrees of the other parameters were graded as follows: 0, none; 1, slight; 2, mild; 3, moderate; and 4, remarkable.

### Subject self‐assessments

Participants were asked to answer a questionnaire comprised of 20 questions about conditions of skin, hair, nail and defecation to assess subjective symptoms using VAS. As shown in Table S2, possible scores ranged 0–100.

We also analyzed subjects’ emotional states using POMS, which was used to assess transient distinct mood states. The original form of POMS was translated into Japanese and a brief version was developed, comprising 30 items.^20^

### Safety monitoring

All subjects were monitored during the study for adverse events and side‐effects. Safety monitoring comprised a daily record and doctor’s inquiries on the general health and occurrence of any health‐related events. The association of adverse events with test food ingestion was determined by the physician based on records while remaining blinded to group allocation. The severity of adverse effects was evaluated according to the Common Terminology Criteria for Adverse Events version 4.0 JCOG/JSCO.

### Sample size

Sample size was calculated according to the previous study.^18^ Because the changes of skin hydration at 12 weeks after *Aloe* sterol intake was estimated to be 4.0 with standard deviation (SD) of 5.5, the sample size required to detect a mean skin hydration change at α = 0.05 and power = 0.90 by unpaired *t*‐test was calculated at 41 study participants per group, totaling 82 study participants. The target sample size was estimated using JMP 13 (SAS Institute, Cary, NC, USA). Considering a 30% dropout rate, approximately 60 participants per group would need to be recruited.

### Statistical analysis

Statistical analysis was performed using SAS version 9.4 (SAS Institute), and was based on the intention‐to‐treat population, which included all randomly assigned participants with at least one observation. All data are presented as means ± SD. The baseline measurements between groups were assessed by an unpaired *t*‐test. Skin hydration levels, TEWL and collagen score were evaluated using ancova with the treatment effect and baseline values as covariates. In a separate analysis, an unpaired *t*‐test was used for intergroup comparisons of the changes of values at each time point (weeks 4, 8 and 12), while a paired *t*‐test was used for intragroup comparisons between week 0 (baseline) and each subsequent time point.

Although not planned before the study initiation, skin hydration data were also analyzed by dividing into subgroups according to baseline levels. Safety analyses were based on summary listings of adverse events.

All analyses were two‐tailed, and *P* < 0.05 was considered significant.

## Results

### Subject demographics

**Figure 1:**
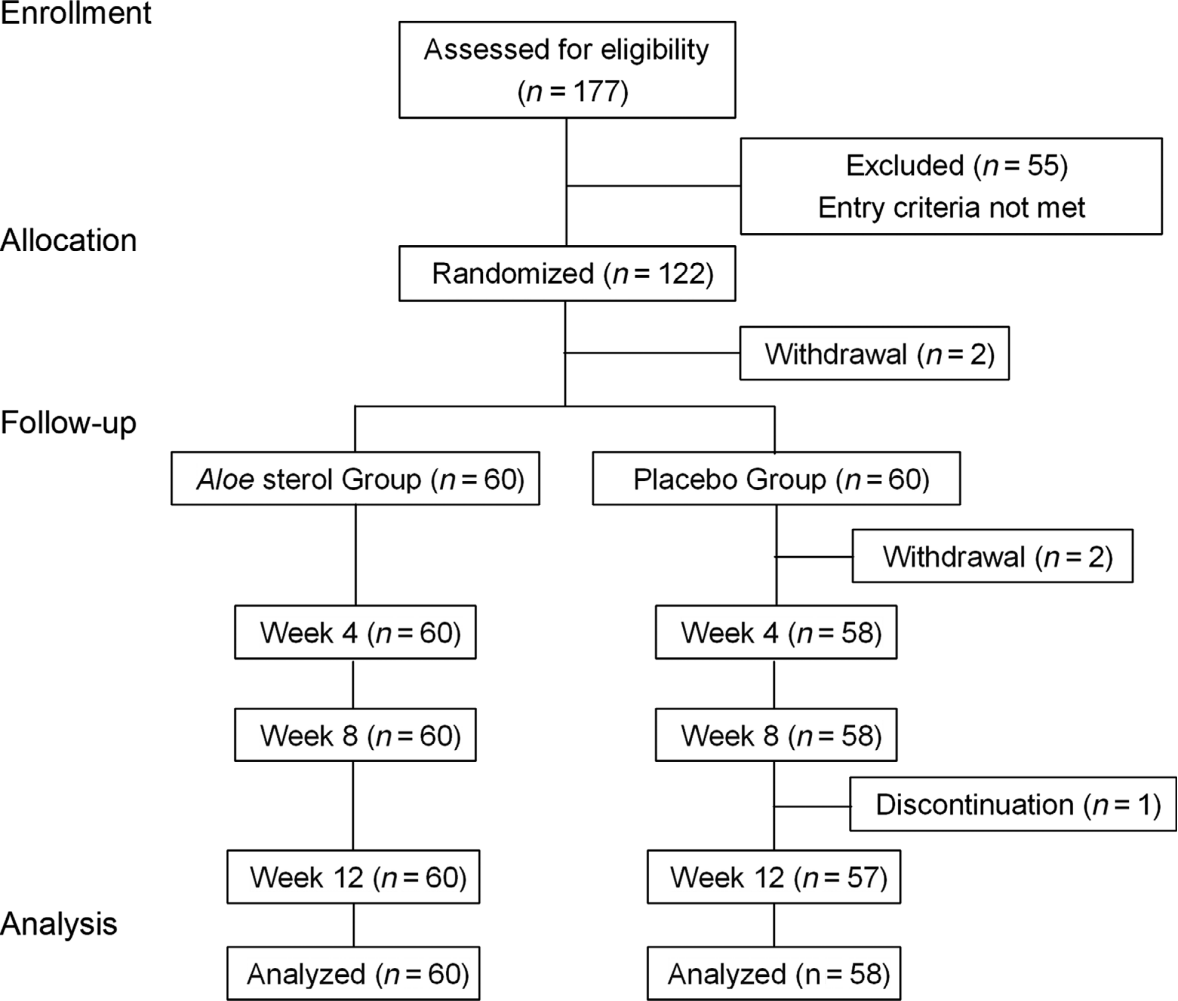
Flow diagram of participants.

According to the inclusion criteria, 177 healthy women were initially enrolled into the study (Table 1). Among them, 55 women were excluded mainly because they used cosmetology or food affecting the skin condition or were judged to be unsuitable (e.g. compliance violations) (Table S1). The remaining 122 participants were randomized to receive either *Aloe* sterol or placebo in a double‐blind manner. Before the test food intake period (week 0), two participants withdrew their consent (Fig. 1). One participant in the placebo group also retracted consent before week 4, and one more participant was considered to be ineligible for inclusion due to poor compliance. One participant also discontinued the study because of enteritis between week 8 and 12.

As a result, we obtained data of 60 participants in the *Aloe* sterol group and 58 in the placebo group. The baseline characteristics (age, height, bodyweight, body mass index and body fat) of the two groups were comparable (Table S3).

### Skin hydration and TEWL

**Figure 2:**
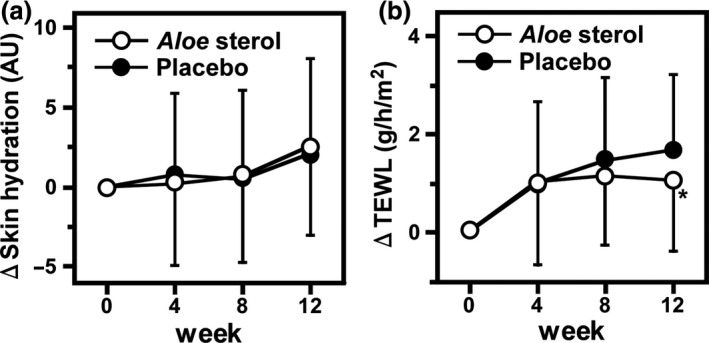
(a) The Δchanges in skin hydration and (b) transepidermal water loss (TEWL) during the treatment period. Data are expressed as means ± standard deviation. (*Aloe* sterol group, *n* = 60; placebo group, *n* = 58). *P < 0.05, versus values in placebo group. AU, arbitrary units.

Skin moisture condition was compared by measuring forearm skin hydration (epidermal hydration) and TEWL (epidermal barrier function) in the *Aloe* sterol and placebo groups. Their basal levels at week 0 were not significantly different between the two groups (Table 2). Skin hydration levels tended to be higher during the test period than baseline in both groups, probably due to seasonal factors: those of the *Aloe* sterol group and placebo group at week 12 (27.98 ± 5.69, *P* = 0.0006, and 27. 78 ± 6.42, *P* = 0.0038, respectively) were significantly greater than at baseline (25.42 ± 4.55 and 25.71 ± 4.64, respectively). However, no significant difference was found in the Δchanges between the two groups throughout the test period (Fig. 2a).

**Table 2:** Comparison of skin hydration levels and TEWL

Parameter	Group	Week	*n*	Mean	SD	*P* ^1^	*P* ^2^
Skin hydration, AU	*Aloe* sterol	0	60	25.42	4.55		
	Placebo		58	25.71	4.64		
	*Aloe* sterol	4	59	25.40	5.62	0.4092	0.8583
	Placebo		58	26.46	6.18		0.2623
	*Aloe* sterol	8	59	26.09	5.96	0.8765	0.3802
	Placebo		58	26.13	5.80		0.4929
	*Aloe* sterol	12	60	27.98	5.69	0.6488	0.0006
	Placebo		57	27.78	6.42		0.0038
TEWL, g/h per m^2^	*Aloe* sterol	0	60	8.65	1.60		
	Placebo		58	8.75	1.73		
	*Aloe* sterol	4	59	9.69	1.84	0.9893	0.0001
	Placebo		58	9.75	1.87		0.0001
	*Aloe* sterol	8	59	9.77	1.61	0.1599	0.0001
	Placebo		58	10.23	2.00		0.0001
	*Aloe* sterol	12	60	9.72	1.42	0.0090	0.0001
	Placebo		57	10.41	1.78		0.0001

*P* < 0.05 was considered statistically significant. *P* ^1^, ancova; *P* ^2^, Student’s *t*‐test. AU, arbitrary units; SD, standard deviation; TEWL, transepidermal water loss.

On the other hand, TEWL values of the *Aloe* sterol group and placebo group were significantly greater at weeks 4, 8 and 12 (9.69 ± 1.84, *P* < 0.0001, 9.77 ± 1.61, *P* < 0.0001, 9.72 ± 1.42, *P* < 0.0001, and 9.75 ± 1.87, *P* < 0.0001, 10.23 ± 2.00, *P* < 0.0001, 10.41 ± 1.78, *P* < 0.0001, respectively) than at baseline (8.65 ± 1.60 and 8.75 ± 1.73, respectively) (Table 2). However, the values were consistently lower in the *Aloe* sterol group during the test period than in the placebo group, and the *Aloe* sterol group showed significant reduction compared with the placebo group at week 12 (difference, −0.641; 95% confidence interval [CI], −1.120 to −0.162; *P* = 0.0090). Additionally, we also found significant difference in the Δchanges between the two groups at week 12 (*P* = 0.0307, Fig. 2b).

### Ultrasound echogenicity

**Figure 3:**
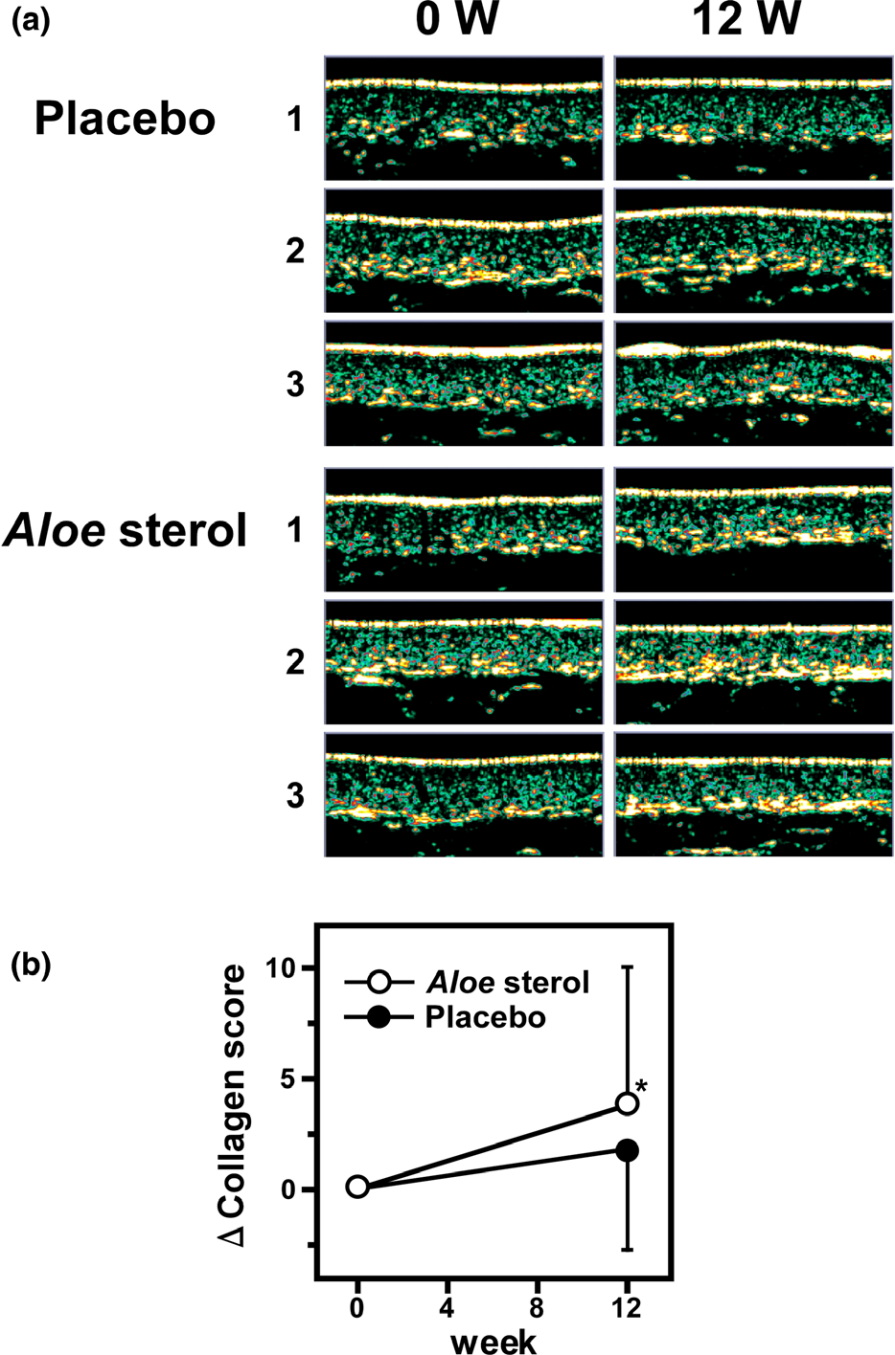
Ultrasonography evaluations. (a) Ultrasound skin images of collagen content before and after 12 weeks of ingestion of *Aloe* sterol or placebo. Representative images of three subjects from each group are shown. (b) ΔChanges in the collagen scores before and after the ingestion. Data are expressed as means ± standard deviation (*Aloe* sterol group, *n* = 60; placebo group, *n* = 58). *P < 0.05, versus values in placebo group.

We examined the condition of dermis by using non‐invasive ultrasonic measurement. Figure 3(a) indicates representative changes in the ultrasound images of six participants (three from each group) before and after 12 weeks of treatment with the *Aloe* sterol supplement or placebo. In the placebo group, high‐intensity echogenicity regions (i.e. high collagen score regions) were similar between weeks 0 and 12. Conversely, we found expansions of high‐intensity echogenicity regions in the images of the *Aloe* sterol group after 12 weeks compared with week 0.

The collagen score measured by ultrasound echography as described in the Methods section are shown in Table 3. Mean collagen scores at week 12 were significantly increased in both the *Aloe* sterol group and placebo group (36.33 ± 4.52, *P* < 0.0001, and 33.64 ± 7.63, *P* = 0.0048, respectively) compared with basal levels at week 0 (32.55 ± 6.94 and 32.20 ± 7.24, respectively). When collagen score was compared between both groups at week 12, the *Aloe* sterol group indicated higher score than the placebo group (difference, 2.159; 95% CI, 0.221–4.097; *P* = 0.0291). Furthermore, the Δchanges at week 12 were also significantly higher in the *Aloe* sterol group than in the placebo group (*P* = 0.0485, Fig. 3b).

**Table 3:** Comparison of collagen scores

Group	Week	*n*	Mean	SD	*P* ^1^	*P* ^2^
*Aloe* sterol	0	60	32.55	6.94		
Placebo		58	32.20	7.24		
*Aloe* sterol	12	60	36.33	7.52	0.0291	<0.0001
Placebo		57	33.64	7.63		0.0048

*P* < 0.05 was considered statistically significant. *P* ^1^, ancova; *P* ^2^, Student’s *t*‐test. SD, standard deviation.

### Observation of skin condition

In facial skin, mean scores of wrinkles and dryness/scales evaluated by a dermatologist were significantly improved both in the *Aloe* sterol group (2.27 ± 1.52, *P* < 0.0001, and 0.05 ± 0.22, *P* < 0.0001, respectively) and the placebo group (2.31 ± 1.61, *P* = 0.0002, and 0.07 ± 0.26, *P* < 0.0001, respectively) at week 12 compared with baseline (2.88 ± 1.77, 0.50 ± 0.68, and 2.74 ± 1.65, 0.55 ± 0.75, respectively) (Table S4). Dryness/scales were also decreased in outer and inner arms of both groups at week 12 (0.22 ± 0.49, *P* < 0.0001, 0.27 ± 0.52, *P* < 0.0001 in *Aloe* sterol group, and 0.11 ± 0.32, *P* < 0.0001, 0.13 ± 0.34, *P* < 0.0001 in placebo group, respectively) compared with those at week 0 (0.90 ± 0.73, 0.63 ± 0.76, and 0.78 ± 0.75, 0.66 ± 0.76, respectively).

On the other hand, significant improvement of facial erythema and pruritus in outer and inner arms at week 12 compared with week 0 was seen only in the *Aloe* sterol group (0.02 ± 0.13 vs 0.08 ± 0.28, *P* = 0.0445, 0.03 ± 0.18 vs 0.22 ± 0.49, *P* = 0.0037, 0.03 ± 0.18 vs 0.13 ± 0.39, *P* = 0.0327), and not in the placebo group (0.02 ± 0.14 vs 0.12 ± 0.42, *P* = 0.0832, 0.06 ± 0.23 vs 0.19 ± 0.54, *P* = 0.0580, 0.04 ± 0.19 vs 0.16 ± 0.52, *P* = 0.4193). Placebo significantly altered only facial papule score at week 12 compared with week 0 (0.02 ± 0.14 vs 0.14 ± 0.40, *P* = 0.0182). *Aloe* sterol also affected the score, but not significantly (0.05 ± 0.22 vs 0.15 ± 0.40, *P* = 0.0832).

### Subjective evaluation

Table S5 shows VAS scores based on a questionnaire of subjective evaluation on various factors including feelings on skin moisture, skin glossiness, tension and texture. Among them, VAS of oily skin at week 12 tended to be higher in the *Aloe* sterol group than placebo group, but not with statistical significance (42.67 ± 20.64 vs 35.33 ± 19.74, *P* = 0.0522).

Significant improvement of VAS of skin acne at week 8 (29.29 ± 29.16, *P* = 0.0003) and week 12 (29.77 ± 27.80, *P* = 0.0019), fingernail brittleness at week 4 (46.75 ± 30.41, *P* = 0.0002), and constipation at week 4 (27.93 ± 27.36, *P* = 0.0120), week 8 (26.76 ± 25.17, *P* = 0.0151) and week 12 (21.77 ± 23.56, *P* = 0.0002) compared with week 0 (40.07 ± 29.96, 59.87 ± 29.62, 34.78 ± 28.80, respectively) was seen only in the *Aloe* sterol group. Meanwhile, only the VAS of rough skin at week 4 was significantly better than baseline in the placebo group (45.67 ± 23.72 vs 52.79 ± 21.29, *P* = 0.0136). We could not find a significant difference in POMS between the two groups (data not shown).

### Subgroup analysis

Taken together, TEWL, collagen score, objective skin condition and subjective symptoms tended to be improved in the *Aloe* sterol group at week 12 compared with the placebo group. However, we did not find statistically significant change in the skin hydration levels between the two groups. Accordingly, although not planned before the study initiation, we performed subgroup analysis focusing on skin moisture.

**Figure 4:**
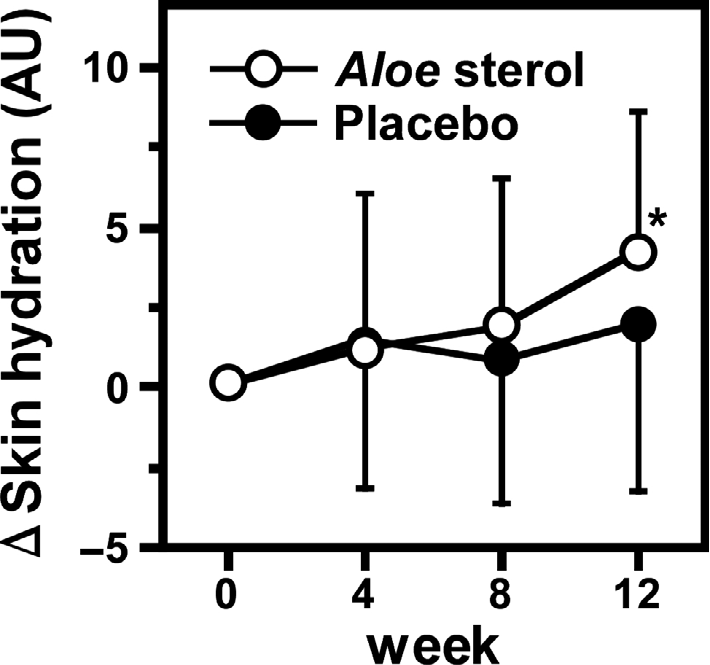
ΔChanges in the skin hydration of participants whose skin hydration was less than 27.4 arbitrary units (AU). Data are expressed as mean ± standard deviation (*Aloe* sterol group, *n* = 38; placebo group, *n* = 34). *P < 0.05, versus values in placebo group.

We supposed that it may be difficult to enhance skin hydration levels in participants with higher basal hydration levels. We defined subjects with already moist skin as those with skin hydration levels more than mean ± standard error (SE) value of participants with 40 μg of *Aloe* sterol for 12 weeks (27.4 arbitrary units [AU]) based on our previous study.^18^ In those without already moist skin, the skin hydration values at week 12 were higher in the *Aloe* sterol group than in the placebo group (26.86 ± 5.19 vs 24.59 ± 4.67; difference, 2.298; 95% CI, 0.093–4.503; *P* = 0.0415) (Table 4), and the Δchange at week 12 was also significantly larger in the *Aloe* sterol group (*P* = 0.0445, Fig. 4).

**Table 4:** Skin hydration levels of the subdivided subjects (skin hydration < 27.4AU)

Parameter	Group	Week	*n*	Mean	SD	*P* ^1^	*P* ^2^
Skin hydration, AU	*Aloe* sterol	0	38	22.64	2.83		
	Placebo		34	22.71	3.14		
	*Aloe* sterol	4	38	23.82	4.67	0.7852	0.1016
	Placebo		34	24.13	4.53		0.0801
	*Aloe* sterol	8	37	24.50	5.08	0.3553	0.0199
	Placebo		34	23.55	4.23		0.2836
	*Aloe* sterol	12	38	26.86	5.19	0.0415	<0.0001
	Placebo		33	24.59	4.67		0.0416

*P* < 0.05 was considered statistically significant. *P* ^1^, ancova; *P* ^2^, Student’s *t*‐test. AU, arbitrary units; SD, standard deviation.

### Adverse events

In the study period (during weeks 0–12）, 240 adverse events were observed (Table 5). Severe adverse effect, enteritis, was seen in one participant. The enteritis was bacterial, however, and was not considered to be treatment‐related.

**Table 5:** Incidence of adverse events

Types of organs	*Aloe* sterol	Placebo
Gastrointestinal abnormalities	23 (17)	28 (16)
General disorders and administration site abnormalities	4 (4)	10 (8)
Infections and infestations	17 (15)	19 (15)
Eye abnormalities	3 (3)	1 (1)
Musculoskeletal and connective tissue abnormalities	8 (7)	2 (2)
Blood and lymphatic system abnormalities	0 (0)	2 (1)
Respiratory, thoracic and mediastinal abnormalities	2 (2)	5 (5)
Ear abnormalities	0 (0)	1 (1)
Injury, poisoning and procedural complications	1 (1)	1 (1)
Nervous system abnormalities	31 (10)	17 (11)
Reproductive system and breast abnormalities	0 (0)	1 (1)
Skin and subcutaneous tissue abnormalities	40 (19)	24 (12)

Data are shown as number of cases (number of subjects).

The frequency of other adverse effects was not significantly different between the *Aloe* sterol group and placebo group. Furthermore, each of them had obvious causes, occurred spontaneously or incidentally, and was judged to be non‐related to *Aloe* sterol.

## DISCUSSION

Our study includes three novel findings. First, TEWL was significantly reduced and collagen score was increased at week 12 in the *Aloe* sterol group compared with the placebo group. The results suggest that low‐dose *Aloe* sterol intake could improve skin barrier function. Additionally, skin elasticity is an important indicator of skin aging and skin condition, which was controlled by dermal collagen content. A previous study analyzed the effect of *A. vera* gel intake on the buttock skin of Korean women using immunostaining and reverse transcription polymerase chain reaction, and found that *A. vera* increases the amount of type I procollagen.^16^ Furthermore, *Aloe* sterol is reported to promote the synthesis of type I and III collagen in cultured human dermal fibroblasts.^17^ Ingested *Aloe* sterol has been shown to reach the peripheral tissues through the bloodstream, so the collagen content of the dermis appears to be induced through the stimulation of fibroblasts by *Aloe* sterol. Low‐dose *Aloe* sterol could improve TEWL levels significantly at 12 weeks of intake as well as high dose.^18^ Conversely, high dose significantly increased skin hydration levels and collagen score quicker (after week 4) than low dose (after week 12). This may be due to the different seasons and subjects between the two studies.

As a limitation of this study, we could not directly evaluate skin elasticity using the Cutometer MPA 580 device (Courage + Khazaka Electronic) due to the inability of the vacuum tube to constantly exert negative pressure to the skin. Thus, the future evaluation of direct effect on skin elasticity and histological examination of skin biopsy specimens would be necessary. However, we supposed that low‐dose *Aloe *sterol supplementation promotes skin elasticity, based on our data of dermal collagen induction.

A second novel finding is that objective assessment of skin condition and the subjective evaluation in participants with *Aloe* sterol was performed for the first time in this study. The significant improvement of objective wrinkles and dryness/scales in both the *Aloe* sterol and placebo group can be explained by the seasonal change, because the current study was performed over winter and spring, as described in the Methods section: skin hydration levels are generally reduced in the winter, and wrinkles and dryness/scales are thought to be affected by skin hydration.

Significant improvement of objective face erythema and pruritus in the inner and outer arms at week 12 compared with week 0 was seen only in the *Aloe* sterol group, not in the placebo group. Among subjective symptoms, significant improvement in VAS of skin acne, fingernail brittleness and constipation was seen only in the *Aloe* sterol group. Fingernail brittleness may be associated with dryness or asteatosis. Acne as well as erythema and pruritus will be worsened by barrier dysfunction through the exacerbation of inflammation. *Aloe* sterol may have additional effects against skin inflammation, erythema, pruritus and acne^21^ through the maintenance of barrier function. Although other parameters correlating dryness or elasticity were not significantly affected, there is a possibility that *Aloe* sterol also has a therapeutic effect against constipation.

Visual analog scale scores were based on subjective symptoms, and there were large variations. On the other hand, objective evaluation of skin condition was based on clinical assessments by a dermatologist, and their expected values should be 0, except for wrinkles that were sometimes worsened by photoaging. Actually, the mean and SD values of each objective parameter except for wrinkles were basically 0 and less than 1, respectively.

A further novel finding of this study is that according to subgroup analysis, in participants with dry skin, the skin hydration values at week 12 were significantly increased in the low‐dose *Aloe* sterol group compared with in the placebo group. Although the analysis was not planned before the study initiation, this result is consistent with that with high‐dose *Aloe* sterol^18^ and our proposal that low‐dose *Aloe* sterol could reduce TEWL levels. We previously demonstrated that *Aloe* sterol stimulates production and synthesis of hyaluronic acid in human dermal fibroblasts.^17^ Furthermore, the hyaluronic acid content in the dermis of mice treated with oral *Aloe* sterol was higher than that of control mice under ultraviolet B irradiation *in vivo*.^22^ In our previous study, we also showed that intake of *Aloe* sterol increases the level of skin hydration as well as the hyaluronic acid synthesis in hairless mice.^22^, ^23^ There is therefore a possibility that *Aloe* sterol may increase the level of skin moisture through promotion of hyaluronic acid composition.

In the current study, the cut‐off level of skin hydration for subgroup analysis was based on the results of our previous study using high‐dose *Aloe* sterol, conducted from summer to winter.^16^ Thus, basal hydration levels in participants of the previous study are expected to be different from those of the current study which was performed over winter and spring. Conversely, basal levels were similar in the two studies, approximately 24–25 AU in the *Aloe* sterol group and placebo group. Accordingly, participants of the current study tended to have already moist skin, and we tried to exclude such subjects to correctly evaluate the effect of low‐dose *Aloe* sterol. We decided to use mean ± SE value of participants with 40 μg of *Aloe* sterol for 12 weeks as the cut‐off, because these subjects are assumed to have elevated levels of skin hydration.

In a recent randomized double‐blind clinical trial on the effects of porcine placenta extract in 45 Japanese healthy subjects conducted from February to April, basal skin hydration levels of arm and Δchange after 8 weeks of intake were 22.07 ± 6.23 and −2.48 ± 1.28 in the placebo group while 19.19 ± 6.32 and 3.38 ± 0.81 in the placenta group, respectively.^24^ Basal TEWL level in another study with eight healthy controls (10.2 ± 2.56) was also similar to that in the current study.^25^ Furthermore, a double‐blind study on the effects of glucosyl ceramide in 50 volunteers with subjective dry skin, arm TEWL levels before and after 8 weeks intake were 8.1 ± 2.0 and 7.9 ± 2.3 in the placebo group while 7.2 ± 1.9 and 8.3 ± 2.7 in the high‐dose ceramide group, respectively.^26^ In addition, according to a questionnaire to rate subjective symptoms based on a 5‐point scale ranging from 1 (good) to 5 (bad), the score of “rough skin” before and after the intake was 2.8 ± 0.6 and 2.2 ± 0.5 in the placebo group whereas 3.2 ± 0.9 and 2.7 ± 1.0 in the ceramide group, respectively. The score of “dull, fragile nail” was 2.6 ± 1.1 and 2.5 ± 1.1 in the placebo group while 3.3 ± 1.3 and 3.0 ± 1.1 in the ceramide group, respectively. Thus, the value of each parameter of our study was consistent with other studies.

Taken together, our results of the double‐blind clinical trial confirmed that daily oral intake of *Aloe* sterol, even at a lower dose (19 μg), significantly increases skin barrier function and skin moisture by improving TEWL and skin hydration level, leading to skin wellness. Ultrasonographic results suggest that intake of *Aloe* sterol increased the collagen content in the dermis. It is considered that the dermal collagen affects skin viscoelasticity which contributes to support of cutaneous structures and protection against injury such as skin tears, and plays important roles in maintenance of healthy tough skin.^27^, ^28^ *Aloe* sterol also affected various objective and subjective symptoms. Histological analysis should be performed in an increased number of participants with a wide age range including males to elucidate the underlying mechanisms of the effects of *Aloe* sterol intake on skin health.

## CONFLICT OF INTEREST

None declared.

## Supporting information


**Table S1.** Reasons for exclusion
**Table S2.** Questions for subject self‐assessments (visual analog scale)
**Table S3.** Baseline characteristics of subjects
**Table S4.** Observation of skin condition
**Table S5.** Visual analog scale scores

## References

[1] Verdier‐Sevrain S , Bonte F . Skin hydration: a review on its molecular mechanisms. J Cosmet Dermatol 2007; 6: 75–82. 17524122 10.1111/j.1473-2165.2007.00300.x

[2] Baroni A . Epidermal barrier function: clinical implications and therapeutics. Clin Dermatol 2012; 30: 255–366. 22507036 10.1016/j.clindermatol.2011.08.029

[3] Brandner JM . Importance of tight junction in relation to skin barrier function. Curr Probl Dermatol 2016; 49: 27–37. 26844895 10.1159/000441541

[4] Tsunemi Y , Nakagami G , Takehara K *et al.* Effects of skin care education for care staff at elderly care facilities on skin conditions of the residents. J Dermatol 2020; 47: 327–333. 31912569 10.1111/1346-8138.15213 PMC7186817

[5] Kamo A , Umehara Y , Negi O *et al.* Effects of Kakato‐tsurutsuru socks on dry heels in healthy volunteer subjects. J Dermatol 2020; 47: 413–417. 31985094 10.1111/1346-8138.15235

[6] Blichmann CW , Serup J . Assessment of skin moisture. Measurement of electrical conductance, capacitance and trans epidermal water loss. Acta Derm Venereol 1988; 69: 284–290. 2459872

[7] Reynolds T , Dweck AC . Aloe vera leaf gel: a review update. J Ethnopharmacol 1999; 68: 3–37. 10624859 10.1016/s0378-8741(99)00085-9

[8] Rodiguez ER , Martin JD , Romero CD . Aloe vera as functional ingredient in foods. Crit Rev Food Sci Nutr 2010; 50: 305–326. 20301017 10.1080/10408390802544454

[9] Choi S , Chung MH . A review of the relationship between aloe vera components and their biologic effects. Semin Integrat Med 2003; 1: 53–62.

[10] Im SA , Oh ST , Song S *et al.* Identification of optimal molecular size of modified Aloe polysaccharides with maximum immunomodulatory activity. Int Immunopharmacol 2005; 5: 271–279. 15652758 10.1016/j.intimp.2004.09.031

[11] Jettancheawchankit S , Sasithanasate S , Sangvanich P , Banlunara W , Thuyakitpisal P . Acemannan stimulates gingival fibroblast proliferation; expressions of keratinocytes growth factor‐1, vascular endothelial growth factor, and type I collagen; and wound healing. J Pharmacol Sci 2009; 109: 525–531. 19372635 10.1254/jphs.08204fp

[12] Wang Z , Li X , Yang Z , He X , Tu J , Zhang T . Effects of aloesin on melanogenesis in pigmented skin equivalents. Int J Cosmet Sci 2008; 30: 121–130. 18377621 10.1111/j.1468-2494.2008.00432.x

[13] Yimam M , Zhao JZ , Corneliusen B , Pantier M , Brownell LA , Jia Q . UP780, a chromone‐enriched Aloe composition improves insulin sensitivity. Metab Syndr Relat Disord 2013; 11: 267–275. 23573999 10.1089/met.2012.0135

[14] Tanaka M , Misawa E , Ito Y *et al.* Identification of five phytosterols from Aloe vera gel as anti‐diabetic compounds. Biol Pharm Bull 2006; 29: 1418–1422. 16819181 10.1248/bpb.29.1418

[15] Nomaguchi K , Tanaka M , Misawa E *et al.* Aloe vera phytosterols act as ligands for PPAR and improve the expression levels of PPAR target genes in the livers of mice with diet‐induced obesity. Obes Res Clin Pract 2011; 5: 190–201. 10.1016/j.orcp.2011.01.002 24331101

[16] Cho S , Lee S , Lee MJ *et al.* Dietary Aloe vera supplementation improves facial wrinkles and elasticity and it increases the type I procollagen expression in human skin in vivo. Ann Dermatol 2009; 21: 6–11. 20548848 10.5021/ad.2009.21.1.6 PMC2883372

[17] Tanaka M , Misawa E , Yamauchi K , Abe F , Ishizaki C . Effects of plant sterols derived from Aloe vera gel on human dermal fibroblasts in vitro and on skin condition in Japanese women. Clin Cosmet Investig Dermatol 2015; 8: 95–104. 10.2147/CCID.S75441 PMC4345938 25759593

[18] Tanaka M , Yamamoto Y , Misawa E *et al.* Effects of Aloe sterols supplementation on skin elasticity, hydration, and collagen score: A 12‐week double‐blind randomized controlled trial. Skin Pharmacol Physiol 2016; 29: 309–317. 28088806 10.1159/000454718

[19] Tanaka M , Yamada M , Toida T , Iwatsuki K . Safety evaluation of supercritical carbon dioxide extract of aloe vera gel. J Food Sci 2012; 71: 2–9. 10.1111/j.1750-3841.2011.02452.x 22260137

[20] Yokoyama K (ed). Manual of the Japanese Version of Profile of Mood States‐Brief. Tokyo: Kaneko Shobo; 2005.

[21] Misawa E , Tanaka M , Saito M *et al.* Protective effects of Aloe sterols against UVB‐induced photoaging in hairless mice. Photdermatol Photoimmunol Photomed 2017; 33: 101–111. 10.1111/phpp.12286 27995657

[22] Saito M , Tanaka M , Misawa E *et al.* Oral administration of Aloe vera gel powder prevents UVB‐induced decrease in skin elasticity via suppression of over expression of MMPs in hairless mice. Biosci Biotech Biochem 2016; 80: 1416–1424. 10.1080/09168451.2016.1156480 27045316

[23] Yao R , Tanaka M , Misawa E *et al.* Daily ingestion of Aloe vera gel powder containing Aloe sterol prevents skin photoaging in OVX hairless mice. J Food Sci 2016; 81: H2849–H2857. 27732760 10.1111/1750-3841.13527

[24] Kim K , Sung J , Lee H , Ono T , Yonei Y . Effect of a dietary supplement containing porcine placenta extract on skin hydration. A placebo‐controlled, Randomized, double‐blind, clinical study. Jpn Pharmacol Ther 2018; 46: 1023–1034.

[25] Choi SJ , Song MG , Sung WT *et al.* Comparison of transepidermal water loss, capacitance and pH values in the skin between intrinsic and extrinsic atopic dermatitis patients. J Korean Med Sci 2003; 18: 93–96. 12589094 10.3346/jkms.2003.18.1.93 PMC3054999

[26] Hori M , Kishimoto S , Tezuka Y *et al.* Double‐blind study on effects of glucosyl ceramide in beet extract on skin elasticity and fibronectin production in human dermal fibroblasts. Anti‐Aging Med 2010; 7: 129–142.

[27] Everett JS , Sommers MS . Skin viscoelasticity: physiologic mechanisms, measurement issues, and application to nursing science. Biol Re Nurs 2013; 15: 338–346. 10.1177/1099800411434151 PMC3465619 22544517

[28] Koyano Y , Nakagami G , Iizaka S *et al.* Exploring the prevalence of skin tears and skin properties related to skin tears in elderly patients at a long‐term medical facility in Japan. Int Wound J 2016; 13: 189–197. 24674027 10.1111/iwj.12251 PMC7949576

