# Supplementary material for: Effects of low‐dose Aloe sterol supplementation on skin moisture, collagen score and objective or subjective symptoms: 12‐week, double‐blind, randomized controlled trial
Source: J Dermatol. 2020 Jun 8;47(9):998–1006. doi: 10.1111/1346-8138.15428 (PMC7496846; doi:10.1111/1346-8138.15428)
Supplement: Supplementary file 1 — Table S1. Reasons for exclusion Table S2. Questions for subject self‐assessments (visual analog scale) Table S3. Baseline characteristics of subjects Table S4. Observation of skin condition Table S5. Visual analog scale scores [file JDE-47-998-s001.docx]

**Table S1. Reasons for exclusion**

| Reasons for non-eligibility | n |
| --- | --- |
| Regularly use cosmetology or food affecting the skin condition | 12 |
| Skin diseases affecting skin condition | 6 |
| Need to wear masks or to blow their nose due to allergic rhinitis | 3 |
| Serious disorders or histories of serious disorders | 6 |
| Excessively consume alcohol | 5 |
| Judged to be otherwise unsuitable for enrollment by the principal investigator | 18 |
| Withdrew consent | 5 |
| Total | 55 |

**Table S2. Questions for subject self-assessments (VAS)**

| Questions |  | 0 |  | 100 |
| --- | --- | --- | --- | --- |
| 1) Skin moist feeling |  | Very moist |  | Not moist |
| 2) Skin grossiness |  | Very glossy |  | Not glossy |
| 3) Skin tension |  | Very firm |  | Not firm |
| 4) Skin texture |  | Very fine |  | Not fine |
| 5) Crusted skin |  | Not at all |  | Extremely |
| 6) Skin dullness |  | Not at all |  | Extremely |
| 7) Dark circles around eyes |  | Not at all |  | Extremely |
| 8) Oily skin |  | Not at all |  | Extremely |
| 9) Skin dryness |  | Not at all |  | Extremely |
| 10) Skin spot |  | Not at all |  | Extremely |
| 11) Skin wrinkles |  | Not at all |  | Extremely |
| 12) Skin transparency |  | Very transparent |  | Not transparent |
| 13) Rough skin |  | Not at all |  | Extremely |
| 14) Skin acne |  | Not at all |  | Extremely |
| 15) Makeup sitting |  | Good |  | Bad |
| 16) Dryness of elbow or knee |  | Not at all |  | Extremely |
| 17) Fingernail brittleness |  | Not at all |  | Extremely |
| 18) Hair loss |  | Not at all |  | Extremely |
| 19) Hair moist feeling |  | Very moist |  | Not moist |
| 20) Hair elasticity |  | Very elastic |  | Not elastic |
| 21) Constipation |  | Not at all |  | Extremely |

**Table S3.　Baseline characteristics of subjects**

|  | *Aloe* sterol  n = 60 | Placebo  n = 58 | p-value |
| --- | --- | --- | --- |
| Age | 44.03 ± 6.80 | 43.74 ± 6.96 | 0.8182 |
| Height (cm) | 159.6 ± 5.74 | 158.8 ± 4.76 | 0.4432 |
| Body weight (kg) | 56.10 ± 8.75 | 55.35 ± 8.47 | 0.6357 |
| BMI (kg/m^2^) | 22.03 ± 3.09 | 21.94 ± 3.12 | 0.8793 |
| Body fat (%) | 30.46 ± 6.15 | 29.88 ± 6.31 | 0.6108 |

p-value：Student’s t-test.

**Table S4. Observation for skin condition**

| Position | Parameter |  | Group | Mean ± SD | p-value 1 | p-value 2 | |
| --- | --- | --- | --- | --- | --- | --- | --- |
| Face | Wrinkles | Week 0  Week 12 | *Aloe* sterol  Placebo  *Aloe* sterol  Placebo | 2.88 ± 1.77  2.74 ± 1.65  2.27 ± 1.52  2.31 ± 1.61 | 0.6530  0.8956 | < | 0.0001  0.0002 |
|  | Dry/Scales | Week 0  Week 12 | *Aloe* sterol  Placebo  *Aloe* sterol  Placebo | 0.50 ± 0.68  0.55 ± 0.75  0.05 ± 0.22  0.07 ± 0.26 | 0.6952  0.5968 | <  < | 0.0001  0.0001 |
|  | Pruritus | Week 0  Week 12 | *Aloe* sterol  Placebo  *Aloe* sterol  Placebo | 0.12 ± 0.37  0.09 ± 0.28  0.03 ± 0.18  0.06 ± 0.23 | 0.6188  0.5669 |  | 0.0581  0.6589 |
|  | Erythema | Week 0  Week 12 | *Aloe* sterol  Placebo  *Aloe* sterol  Placebo | 0.08 ± 0.28  0.12 ± 0.42  0.02 ± 0.13  0.02 ± 0.14 | 0.5704  0.9407 |  | 0.0445  0.0832 |
|  | Papules | Week 0  Week 12 | *Aloe* sterol  Placebo  *Aloe* sterol  Placebo | 0.15 ± 0.40  0.14 ± 0.40  0.05 ± 0.22  0.02 ± 0.14 | 0.8701  0.3661 |  | 0.0832  0.0182 |
| Outer  Arm | Dry/Scales | Week 0  Week 12 | *Aloe* sterol  Placebo  *Aloe* sterol  Placebo | 0.90 ± 0.73  0.78 ± 0.75  0.22 ± 0.49  0.11 ± 0.32 | 0.3640  0.1804 | <  < | 0.0001  0.0001 |
|  | Pruritus | Week 0  Week 12 | *Aloe* sterol  Placebo  *Aloe* sterol  Placebo | 0.22 ± 0.49  0.19 ± 0.54  0.03 ± 0.18  0.06 ± 0.23 | 0.7774  0.5669 |  | 0.0037  0.0580 |
|  | Erythema | Week 0  Week 12 | *Aloe* sterol  Placebo  *Aloe* sterol  Placebo | 0.05 ± 0.22  0.03 ± 0.26  0.00 ± 0.00  0.00 ± 0.00 | 0.7281  - |  | 0.0832  - |
|  | Papules | Week 0  Week 12 | *Aloe* sterol  Placebo  *Aloe* sterol  Placebo | 0.02 ± 0.13  0.03 ± 0.26  0.00 ± 0.00  0.00 ± 0.00 | 0.6392  - |  | 0.3214  - |
| Inner  Arm | Dry/Scales | Week 0  Week 12 | *Aloe* sterol  Placebo  *Aloe* sterol  Placebo | 0.63 ± 0.76  0.66 ± 0.76  0.27 ± 0.52  0.13 ± 0.34 | 0.8763  0.1007 | <  < | 0.0001  0.0001 |
|  | Pruritus | Week 0  Week 12 | *Aloe* sterol  Placebo  *Aloe* sterol  Placebo | 0.13 ± 0.39  0.16 ± 0.52  0.03 ± 0.18  0.04 ± 0.19 | 0.7970  0.9155 |  | 0.0327  0.4193 |
|  | Erythema | Week 0  Week 12 | *Aloe* sterol  Placebo  *Aloe* sterol  Placebo | 0.05 ± 0.22  0.03 ± 0.26  0.02 ± 0.13  0.00 ± 0.00 | 0.7281  0.3450 |  | 0.3214  - |
|  | Papules | Week 0  Week 12 | *Aloe* sterol  Placebo  *Aloe* sterol  Placebo | 0.02 ± 0.13  0.03 ± 0.26  0.01 ± 0.06  0.02 ± 0.14 | 0.6392  0.6050 |  | 0.6585  0.3219 |

p-value 1: t test, p-value 2: paired t.

Wrinkles: score 0=absence of symptom; score 7=maximum severity.

The others: score 0=absence of symptom; score 4=maximum severity.

**Table S5-1. VAS scores**

| Parameter |  | Group | n | Mean ± SD | p-value 1 | p-value 2 | |
| --- | --- | --- | --- | --- | --- | --- | --- |
| Skin moist feeling | Week 0 | *Aloe* sterol  Placebo | 60  58 | 70.37 ± 13.69  70.79 ± 12.17 | 0.8585 |  |  |
|  | Week 4 | *Aloe* sterol  Placebo | 59  58 | 59.49 ± 15.18  58.02 ± 15.45 | 0.6037 | < | 0.0001  0.0001 |
|  | Week 8 | *Aloe* sterol  Placebo | 59  58 | 55.98 ± 16.74  55.16 ± 17.26 | 0.7927 | <  < | 0.0001  0.0001 |
|  | Week 12 | *Aloe* sterol  Placebo | 60  57 | 50.03 ± 15.94  51.51 ± 16.57 | 0.6244 | <  < | 0.0001  0.0001 |
| Skin glossiness | Week 0 | *Aloe* sterol  Placebo | 60  58 | 72.50 ± 12.89  71.72 ± 12.67 | 0.7422 |  |  |
|  | Week 4 | *Aloe* sterol  Placebo | 59  58 | 60.29 ± 15.58  59.14 ± 16.74 | 0.7011 | <  < | 0.0001  0.0001 |
|  | Week 8 | *Aloe* sterol  Placebo | 59  58 | 57.22 ± 18.03  56.55 ± 16.97 | 0.8368 | <  < | 0.0001  0.0001 |
|  | Week 12 | *Aloe* sterol  Placebo | 60  57 | 51.48 ± 17.25  52.42 ± 16.42 | 0.7640 | <  < | 0.0001  0.0001 |
| Skin tension | Week 0 | *Aloe* sterol  Placebo | 60  58 | 73.10 ± 14.39  74.00 ± 16.07 | 0.7490 |  |  |
|  | Week 4 | *Aloe* sterol  Placebo | 59  58 | 61.68 ± 17.16  58.47 ± 18.09 | 0.3265 | <  < | 0.0001  0.0001 |
|  | Week 8 | *Aloe* sterol  Placebo | 59  58 | 58.42 ± 15.72  59.79 ± 16.24 | 0.6439 | <  < | 0.0001  0.0001 |
|  | Week 12 | *Aloe* sterol  Placebo | 60  57 | 52.38 ± 15.61  54.61 ± 17.42 | 0.4667 | <  < | 0.0001  0.0001 |
| Skin texture | Week 0 | *Aloe* sterol  Placebo | 60  58 | 69.90 ± 16.71  71.52 ± 18.79 | 0.6219 |  |  |
|  | Week 4 | *Aloe* sterol  Placebo | 59  58 | 58.25 ± 18.86  59.88 ± 18.36 | 0.6377 | <  < | 0.0001  0.0001 |
|  | Week 8 | *Aloe* sterol  Placebo | 59  58 | 57.59 ± 17.64  56.91 ± 20.15 | 0.8464 | <  < | 0.0001  0.0001 |
|  | Week 12 | *Aloe* sterol  Placebo | 60  57 | 53.95 ± 17.37  55.28 ± 18.59 | 0.6897 | <  < | 0.0001  0.0001 |
| Crusted skin | Week 0 | *Aloe* sterol  Placebo | 60  58 | 76.63 ± 15.83  78.17 ± 15.56 | 0.5954 |  |  |
|  | Week 4 | *Aloe* sterol  Placebo | 59  58 | 67.71 ± 19.15  64.97 ± 19.13 | 0.4394 | < | 0.0053  0.0001 |
|  | Week 8 | *Aloe* sterol  Placebo | 59  58 | 62.69 ± 21.84  62.59 ± 22.06 | 0.9787 | < | 0.0001  0.0001 |
|  | Week 12 | *Aloe* sterol  Placebo | 60  57 | 52.33 ± 22.64  57.44 ± 22.12 | 0.2201 | <  < | 0.0001  0.0001 |

**Table S5-2. VAS scores**

| Parameter |  | Group | n | Mean ± SD | p-value 1 | p-value 2 | |
| --- | --- | --- | --- | --- | --- | --- | --- |
| Skin dullness | Week 0 | *Aloe* sterol  Placebo | 60  58 | 72.22 ± 15.76  73.98 ± 16.00 | 0.5469 |  |  |
|  | Week 4 | *Aloe* sterol  Placebo | 59  58 | 64.78 ± 17.03  62.43 ± 18.02 | 0.4702 | < | 0.0066  0.0001 |
|  | Week 8 | *Aloe* sterol  Placebo | 59  58 | 61.25 ± 17.90  60.57 ± 20.12 | 0.8459 | < | 0.0001  0.0001 |
|  | Week 12 | *Aloe* sterol  Placebo | 60  57 | 56.73 ± 17.35  58.91 ± 20.42 | 0.5345 | <  < | 0.0001  0.0001 |
| Dark circles around eyes | Week 0 | *Aloe* sterol  Placebo | 60  58 | 67.45 ± 24.45  64.09 ± 23.85 | 0.4510 |  |  |
|  | Week 4 | *Aloe* sterol  Placebo | 59  58 | 58.63 ± 23.48  52.48 ± 23.78 | 0.1623 | < | 0.0048  0.0001 |
|  | Week 8 | *Aloe* sterol  Placebo | 59  58 | 57.59 ± 25.02  54.72 ± 24.05 | 0.5285 |  | 0.0016  0.0001 |
|  | Week 12 | *Aloe* sterol  Placebo | 60  57 | 53.05 ± 24.58  53.86 ± 23.84 | 0.8569 | < | 0.0001  0.0004 |
| Oily skin | Week 0 | *Aloe* sterol  Placebo | 60  58 | 39.20 ± 22.49  35.50 ± 20.30 | 0.3507 |  |  |
|  | Week 4 | *Aloe* sterol  Placebo | 59  58 | 35.92 ± 18.29  34.24 ± 20.26 | 0.6398 |  | 0.1079  0.6020 |
|  | Week 8 | *Aloe* sterol  Placebo | 59  58 | 38.34 ± 20.47  37.76 ± 20.89 | 0.8796 |  | 0.8180  0.4396 |
|  | Week 12 | *Aloe* sterol  Placebo | 60  57 | 42.67 ± 20.64  35.33 ± 19.74 | 0.0522 |  | 0.1571  0.8303 |
| Skin dryness | Week 0 | *Aloe* sterol  Placebo | 60  58 | 79.18 ± 12.20  80.21 ± 11.14 | 0.6353 |  |  |
|  | Week 4 | *Aloe* sterol  Placebo | 59  58 | 68.88 ± 15.10  70.57 ± 12.76 | 0.5155 | < | 0.0001  0.0001 |
|  | Week 8 | *Aloe* sterol  Placebo | 59  58 | 65.61 ± 18.99  66.41 ± 19.72 | 0.8227 | <  < | 0.0001  0.0001 |
|  | Week 12 | *Aloe* sterol  Placebo | 60  57 | 56.10 ± 21.41  60.84 ± 20.66 | 0.2257 | <  < | 0.0001  0.0001 |
| Skin spot | Week 0 | *Aloe* sterol  Placebo | 60  58 | 77.20 ± 16.48  73.90 ± 18.54 | 0.3080 |  |  |
|  | Week 4 | *Aloe* sterol  Placebo | 59  58 | 67.81 ± 18.68  68.62 ± 17.91 | 0.8119 | < | 0.0001  0.0026 |
|  | Week 8 | *Aloe* sterol  Placebo | 59  58 | 69.02 ± 17.93  66.69 ± 19.55 | 0.5034 | < | 0.0001  0.0026 |
|  | Week 12 | *Aloe* sterol  Placebo | 60  57 | 65.52 ± 19.67  64.12 ± 22.43 | 0.7211 | < | 0.0001  0.0004 |

**Table S5-3. VAS scores**

| Parameter |  | Group | n | Mean ± SD | p-value 1 | p-value 2 | |
| --- | --- | --- | --- | --- | --- | --- | --- |
| Skin wrinkles | Week 0 | *Aloe* sterol  Placebo | 60  58 | 68.10 ± 21.86  69.69 ± 18.29 | 0.6697 |  |  |
|  | Week 4 | *Aloe* sterol  Placebo | 59  58 | 62.17 ± 21.88  62.53 ± 18.93 | 0.9233 | < | 0.0076  0.0001 |
|  | Week 8 | *Aloe* sterol  Placebo | 59  58 | 58.53 ± 21.18  63.12 ± 19.86 | 0.2287 | < | 0.0001  0.0006 |
|  | Week 12 | *Aloe* sterol  Placebo | 60  57 | 56.57 ± 22.31  58.70 ± 20.40 | 0.5907 | <  < | 0.0001  0.0001 |
| Skin transparency | Week 0 | *Aloe* sterol  Placebo | 60  58 | 73.28 ± 14.91  73.07 ± 17.15 | 0.9423 |  |  |
|  | Week 4 | *Aloe* sterol  Placebo | 59  58 | 63.93 ± 17.30  63.50 ± 16.70 | 0.8909 | <  < | 0.0001  0.0001 |
|  | Week 8 | *Aloe* sterol  Placebo | 59  58 | 61.81 ± 17.63  60.66 ± 18.28 | 0.7278 | <  < | 0.0001  0.0001 |
|  | Week 12 | *Aloe* sterol  Placebo | 60  57 | 57.83 ± 18.17  58.89 ± 17.00 | 0.7451 | <  < | 0.0001  0.0001 |
| Rough skin | Week 0 | *Aloe* sterol  Placebo | 60  58 | 56.02 ± 23.82  52.79 ± 21.29 | 0.4404 |  |  |
|  | Week 4 | *Aloe* sterol  Placebo | 59  58 | 49.17 ± 24.55  45.67 ± 23.72 | 0.4350 |  | 0.0793  0.0136 |
|  | Week 8 | *Aloe* sterol  Placebo | 59  58 | 46.54 ± 22.77  40.57 ± 20.70 | 0.1406 | < | 0.0091  0.0001 |
|  | Week 12 | *Aloe* sterol  Placebo | 60  57 | 42.98 ± 23.99  38.74 ± 21.16 | 0.3130 | <  < | 0.0001  0.0001 |
| Skin acne | Week 0 | *Aloe* sterol  Placebo | 60  58 | 40.07 ± 29.96  32.64 ± 24.48 | 0.1437 |  |  |
|  | Week 4 | *Aloe* sterol  Placebo | 59  58 | 34.75 ± 28.40  30.97 ± 22.54 | 0.4273 |  | 0.1259  0.5526 |
|  | Week 8 | *Aloe* sterol  Placebo | 59  58 | 29.29 ± 29.16  33.34 ± 22.79 | 0.3734 |  | 0.0003  0.8152 |
|  | Week 12 | *Aloe* sterol  Placebo | 60  57 | 29.77 ± 27.80  29.18 ± 23.26 | 0.9012 |  | 0.0019  0.2196 |
| Makeup sitting | Week 0 | *Aloe* sterol  Placebo | 60  58 | 67.53 ± 15.67  66.53 ± 17.10 | 0.7412 |  |  |
|  | Week 4 | *Aloe* sterol  Placebo | 59  58 | 52.59 ± 15.98  54.69 ± 16.07 | 0.4807 | <  < | 0.0001  0.0001 |
|  | Week 8 | *Aloe* sterol  Placebo | 59  58 | 48.80 ± 16.37  51.64 ± 18.12 | 0.3752 | <  < | 0.0001  0.0001 |
|  | Week 12 | *Aloe* sterol  Placebo | 60  57 | 47.93 ± 19.48  46.39 ± 20.45 | 0.6759 | <  < | 0.0001  0.0001 |

**Table S5-4. VAS scores**

| Parameter |  | Group | n | Mean ± SD | p-value 1 | p-value 2 | |
| --- | --- | --- | --- | --- | --- | --- | --- |
| Dryness of elbow or knee | Week 0 | *Aloe* sterol  Placebo | 60  58 | 78.42 ± 15.02  78.09 ± 12.58 | 0.8973 |  |  |
|  | Week 4 | *Aloe* sterol  Placebo | 59  58 | 65.64 ± 18.95  68.48 ± 18.27 | 0.4114 | < | 0.0001  0.0002 |
|  | Week 8 | *Aloe* sterol  Placebo | 59  58 | 61.14 ± 20.88  62.53 ± 20.46 | 0.7151 | <  < | 0.0001  0.0001 |
|  | Week 12 | *Aloe* sterol  Placebo | 60  57 | 51.37 ± 25.70  56.28 ± 24.32 | 0.2908 | <  < | 0.0001  0.0001 |
| Fingernail brittleness | Week 0 | *Aloe* sterol  Placebo | 60  58 | 59.87 ± 29.62  57.98 ± 29.47 | 0.7297 |  |  |
|  | Week 4 | *Aloe* sterol  Placebo | 59  58 | 46.75 ± 30.41  52.45 ± 29.00 | 0.3016 |  | 0.0002  0.1253 |
|  | Week 8 | *Aloe* sterol  Placebo | 59  58 | 44.36 ± 32.21  46.22 ± 28.40 | 0.7401 | < | 0.0001  0.0018 |
|  | Week 12 | *Aloe* sterol  Placebo | 60  57 | 39.22 ± 30.12  46.88 ± 30.02 | 0.1712 | < | 0.0001  0.0042 |
| Hair loss | Week 0 | *Aloe* sterol  Placebo | 60  58 | 65.35 ± 24.52  62.41 ± 28.93 | 0.5527 |  |  |
|  | Week 4 | *Aloe* sterol  Placebo | 59  58 | 53.19 ± 27.55  54.29 ± 24.49 | 0.8189 |  | 0.0029  0.0081 |
|  | Week 8 | *Aloe* sterol  Placebo | 59  58 | 54.10 ± 25.95  52.34 ± 24.56 | 0.7076 |  | 0.0094  0.0041 |
|  | Week 12 | *Aloe* sterol  Placebo | 60  57 | 46.22 ± 28.46  46.88 ± 26.25 | 0.8966 | <  < | 0.0001  0.0001 |
| Hair moist feeling | Week 0 | *Aloe* sterol  Placebo | 60  58 | 72.90 ± 14.81  74.19 ± 17.78 | 0.6690 |  |  |
|  | Week 4 | *Aloe* sterol  Placebo | 59  58 | 63.76 ± 19.67  63.52 ± 21.47 | 0.9487 |  | 0.0006  0.0002 |
|  | Week 8 | *Aloe* sterol  Placebo | 59  58 | 60.41 ± 21.69  60.31 ± 22.48 | 0.9812 | <  < | 0.0001  0.0001 |
|  | Week 12 | *Aloe* sterol  Placebo | 60  57 | 51.18 ± 23.86  58.63 ± 22.60 | 0.0860 | <  < | 0.0001  0.0001 |
| Hair elasticity | Week 0 | *Aloe* sterol  Placebo | 60  58 | 64.83 ± 20.31  59.10 ± 24.59 | 0.1696 |  |  |
|  | Week 4 | *Aloe* sterol  Placebo | 59  58 | 54.44 ± 23.35  50.57 ± 23.11 | 0.3693 |  | 0.0001  0.0072 |
|  | Week 8 | *Aloe* sterol  Placebo | 59  58 | 56.03 ± 23.60  52.83 ± 25.73 | 0.4837 |  | 0.0055  0.0348 |
|  | Week 12 | *Aloe* sterol  Placebo | 60  57 | 47.82 ± 23.31  49.74 ± 24.55 | 0.6652 | < | 0.0001  0.0025 |

**Table S5-5. VAS scores**

| Parameter |  | Group | n | Mean ± SD | p-value 1 | p-value 2 | |
| --- | --- | --- | --- | --- | --- | --- | --- |
| Constipation | Week 0 | *Aloe* sterol  Placebo | 60  58 | 34.78 ± 28.80  30.76 ± 29.20 | 0.4525 |  |  |
|  | Week 4 | *Aloe* sterol  Placebo | 59  58 | 27.93 ± 27.36  27.16 ± 24.89 | 0.8727 |  | 0.0120  0.2744 |
|  | Week 8 | *Aloe* sterol  Placebo | 59  58 | 26.76 ± 25.17  26.50 ± 22.40 | 0.9526 |  | 0.0151  0.1853 |
|  | Week 12 | *Aloe* sterol  Placebo | 60  57 | 21.77 ± 23.56  25.91 ± 24.29 | 0.3507 |  | 0.0002  0.2008 |

p-value 1: t test, p-value 2: paired t.

Definition of each parameter is described in Table S2.
